# Early High-Dose Caffeine Improves Respiratory Outcomes in Preterm Infants

**DOI:** 10.3390/children8060501

**Published:** 2021-06-13

**Authors:** Vineet Lamba, Oscar Winners, Prem Fort

**Affiliations:** 1 Department of Pediatrics, University of Tennessee Health Sciences Center, Memphis, TN 38103, USA; 2 Johns Hopkins All Children’s Maternal Fetal and Neonatal Institute, Johns Hopkins All Children’s Hospital, St. Petersburg, FL 33701, USA; owinner1@jhmi.edu; 3 Department of Pediatrics, Division of Neonatology, Johns Hopkins University School of Medicine, Baltimore, MD 21205, USA; pfort1@jhmi.edu

**Keywords:** caffeine, apnea of prematurity, bronchopulmonary dysplasia, prematurity

## Abstract

The objective of the study is to determine if early high-dose caffeine (HD) therapy is associated with shorter duration of mechanical ventilation, bronchopulmonary dysplasia (BPD), or decreased need for mechanical ventilation. We conducted a single center, retrospective cohort study of 273 infants less than 32 weeks gestational age (GA). Infants receiving early HD (10 mg/kg/day maintenance) caffeine citrate started within 24 h of life were compared with those receiving LD (6 mg/kg/day) with variable timing of initiation using linear and logistic regression models. The infants in the early HD group had 91.4 (95% confidence interval (CI): −166.6, −16.1; *p* = 0.018) less hours of mechanical ventilation up to 36 weeks PMA or discharge as compared with the LD group. Moreover, infants in the HD group had 0.37 (95% CI: 0.14, 0.97; *p* = 0.042) times lower odds of developing moderate/severe BPD compared with the LD group. Infants receiving early HD caffeine had improved respiratory outcomes with no increase in measured comorbidities. Large prospective studies are needed to determine the long-term outcomes of using high-dose caffeine prophylaxis for preterm infants.

## 1. Introduction

Caffeine citrate is one of the most widely used medications in the neonatal intensive care unit (NICU) [1]. It is a methylxanthine class agent, which has been well validated as a treatment for apnea of prematurity (AoP) [2,3]. It has also been shown to increase the chances of successful extubation [4]. Proposed mechanisms include enhancement of respiratory control [5], and improvement in diaphragmatic contractility and airway function [6,7].

A significant number of extremely low birth weight (ELBW) infants require prolonged mechanical ventilation [8,9], which is associated with poor neurodevelopmental outcomes and bronchopulmonary dysplasia (BPD) [8]. The Caffeine for Apnea of Prematurity (CAP) trial showed that caffeine administration within the first 10 days of life for AoP prevention resulted in a reduction in time on positive pressure ventilation by one week, as well as lowering the incidence of BPD (OR 0.64; 95% confidence interval [CI], 0.52–0.78; *p* ≤ 0.001) [10]. Moreover, long term follow-up revealed improved rates of survival without neurodevelopmental disability at 18 to 21 months (OR 0.77; 95% CI, 0.64–0.93; *p* = 0.008) [11]. Results from a post-hoc subgroup analysis of the CAP trial showed that postnatal age at the time that treatment was initiated may influence the effect of caffeine. Specifically, BPD was reduced by 52% in infants whose treatment was started on postnatal days one–three (early) compared with a 23% reduction in those started after day three (late) [12].

Additional data from the Pediatrix network [13] and the Canadian Neonatal Network [14] demonstrated that earlier initiation of caffeine showed reduced incidence of BPD and a shorter duration of mechanical ventilation. Higher doses of caffeine than historically used as standard in NICUs has sparked interest. A recent systematic review suggested that a higher dose of caffeine (10 to 30 mg/kg/day of caffeine citrate) may enhance its beneficial effect on BPD [15]. However, there was a significant variability in the indication for caffeine use and its time of initiation.

While there has been a shift towards early initiation of caffeine, the efficacy and safety of prophylactic use and higher dosing of caffeine is still not well established. Our study aims to evaluate the effect of this prophylactic initiation of high-dose caffeine citrate (10 mg/kg/day maintenance dose) on the respiratory outcomes in premature infants.

## 2. Materials and Methods

### 2.1. Patient Population

This retrospective cohort study was conducted at a single, regional referral 97-bed level IV NICU (Johns Hopkins All Children’s Hospital). A clinical practice guideline (CPG) related to caffeine administration changed in January 2016. Prior to the CPG, infants were treated with caffeine at the onset of AoP, with a maintenance caffeine citrate dose of 6 mg/kg/day. After the CPG, all infants born at less than 32 weeks gestational age were started on a maintenance dose of 10 mg/kg/day at birth or admission, and then dosing was weight-adjusted weekly until discontinuation. The loading dose remained unchanged at 20 mg/kg/day and the recommendation in the CPG was to continue caffeine to 34–35 wk PMA, or until infants no longer had apneic events off NIV or high-flow respiratory support for five days. The study population included eligible infants admitted to the NICU one-year pre- and post-CPG, i.e., between January 2015 and February 2017. Primary inclusion criteria were: (1) birth gestational age (GA) less than 32 completed weeks and (2) admission to the NICU within seven days of life. Exclusion criteria were: (1) congenital or chromosomal anomalies, (2) death within 24 h of birth, (3) incomplete data in the chart. Secondarily, birth weight (BW) > 2200 g (90th percentile for 32 weeks) was added as an additional exclusion due to some infants being of BW several standard deviations above the normal for their assigned gestation, likely due to an error in charting the GA. Infants who met these criteria were divided into one of two groups based on the dose and timing of their first maintenance dose of caffeine citrate: early high-dose group and low-dose group. Due to a lag in the adoption of the CPG during our study epoch, infants in the post-CPG epoch continued to receive a low caffeine dose for some time. Hence, we performed the analysis by allocating patients in a 2:1 proportion of LD to HD.

An electronic medical record database (Cerner, Cerner Corporation, North Kansas City, MO, USA) was queried to generate a list of all infants meeting the inclusion criteria. Further data were obtained by individual chart review. Institutional review board (IRB) approval was obtained from the Johns Hopkins University IRB, and the hospital Maternal, Fetal & Neonatal Institute research committee provided oversight. This study protocol was reviewed and approved by the Johns Hopkins Medicine Institutional Review Board on 24 August 2017 (IRB approval number IRB00135121). The study was approved for the inclusion of children with permission of parents/guardians waived as ‘research not involving greater than minimal risk’.

### 2.2. Definitions

For the purpose of this study, infants were assigned to early high-dose (HD) and low-dose (LD) groups based on their initial maintenance dose and timing of caffeine citrate—10 mg/kg/day maintenance with loading dose given less than 24 h of life and 6 mg/kg/day with variable timing of loading dose, respectively.

Outcomes were compared between the low-dose (6 mg/kg/day) and the early high-dose (10 mg/kg/day) groups. The primary outcome was the duration of mechanical ventilation up to 36 weeks postmenstrual age (PMA) or discharge. Other outcomes included the need for mechanical ventilation in the first 28 days of life, the duration of non-invasive ventilation up to 36 weeks PMA, the incidence of BPD (including severity subtypes), the number of significant apneas up to 36 weeks PMA, and the length of hospitalization. We assessed the prevalence of major co-morbidities, such as necrotizing enterocolitis (NEC) and retinopathy of prematurity (ROP).

Mechanical ventilation was defined as being intubated and receiving mechanical ventilation, and the duration of mechanical ventilation was determined by the total hours of mechanical ventilation until 36 weeks PMA or discharge, based on Electronic Medical Record (EMR) query. BPD and its severity were defined based on the NICHD consensus definition [16], with need for supplemental oxygen and/or positive pressure at ≥28 days of life and then 36 weeks PMA or discharge, as determined by chart review. We considered any flow > 2 L per minute as positive pressure. Significant apneas were defined as documentation of a witnessed apneic event with desaturation requiring stimulation and/or positive pressure ventilation per EMR query. Gestational age (GA) was determined by the documentation in EMR. Presence of PDA was determined by review of echocardiogram results and surgical ligation by review of operative history. Maternal chorioamnionitis was based on obstetrician-assigned diagnosis based on maternal fever and symptoms. Antenatal steroids were defined as any antenatal betamethasone administration. Postnatal steroid use was defined as the receipt of dexamethasone for evolving BPD which was typically administered as a cumulative dose of 0.89 mg/kg over 10 days per institutional guideline. The diagnoses of NEC and ROP were based on ICD diagnostic codes documented in the patient record. Mortality was defined for all patients who died prior to discharge.

### 2.3. Statistical Analysis

All statistical analyses were performed using SAS 9.4 (SAS Institute Inc., Cary, NC, USA), with a *p*-value of <0.05 considered statistically significant. Statistical significance for unadjusted comparisons was performed using independent samples *t*-tests, Mann–U Whitney, Chi-square, or Fisher’s exact based on the distribution. For outcomes, linear and logistic regression analysis with adjustment for covariates that are evidence-based predictors of the outcome of interest were included in separate models fit to the primary and secondary outcomes. We constructed the following models:

1. For the outcome of duration of mechanical ventilation up to 36 weeks PMA, the following evidence-based co-variates were included in the model: GA [17], BW [17], gender [17], and postnatal steroids [18]. However, birthweight was excluded from the model due to multi-collinearity and model fit. Additionally, PDA was considered to be a potential confounder. PDA was included in the final model based on the results of a partial F-test, increased in R-squared (accounting for variability), and statistical significance within the adjusted model.
2. The following evidence-based co-variates were included in the model for the outcome of the need for mechanical ventilation in the first 28 days of life: GA [19], BW [19], mode of delivery [20], gender [19], Apgar score at 5 min [21], and antenatal steroids [22]. However, birthweight and Apgar score at 5 min were excluded from the model due to multi-collinearity and model fit. Additionally, PDA was also considered to be a potential confounder a priori and was included in the final model based on the results of a likelihood ratio test and statistical significance within the adjusted model.
3. The model examining the relationship between BPD and caffeine groups included the following evidence-based co-variates: GA [17], BW [17], gender [17], chorioamnionitis [23], and postnatal steroids [18]. However, GA and postnatal steroids were excluded from the model due to multi-collinearity and model fit. Additionally, PDA was also considered to be a potential confounder a priori and was included in the final model based on the results of a likelihood ratio test and statistical significance within the adjusted model.

The relationship between caffeine dosing and mild, moderate, and severe BPD was assessed using the Mantel Haenszel Chi-square test, adjusting for modified Ridit scoring, as we cannot assume that BPD categories are equally spaced. Upon model assumption evaluation for proportional odds regression, the proportional odds assumption was not satisfied. As a result, we constructed two models, one where BPD was dichotomized into BPD and no BPD, and the other, which included no BPD/mild BPD vs. moderate/severe BPD as the latter is clinically relevant. Both models were adjusted for similar co-variates as described above.

## 3. Results

**Figure 1:**
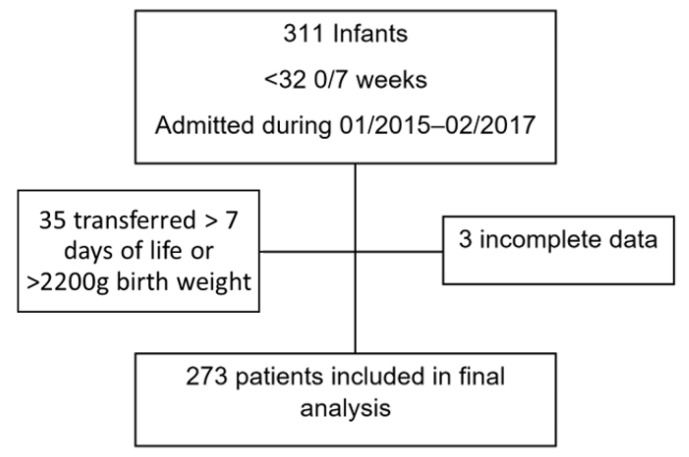
Study population.

During the two-year study period, we identified 311 infants less than 32 weeks GA who were treated with caffeine citrate. Of this population, 38 infants were excluded, 3 due to missing information and an additional 35 patients due to birth weight > 2200 g and/or transferred from outside facilities at >7 days of life (DOL) (Figure 1). The infants in the HD group all received caffeine citrate within 24 h of birth per CPG, while, in the LD group, infants had variable timing of caffeine administration. All infants in the HD group were admitted in the post-CPG epoch (February 2016–February 2017). However, the LD were seen in both epochs, likely due to delay in adoption of the guideline. Of the 273 infants included in the final analysis, 67.0% (*n* = 183) were in the LD group and 33% (*n* = 90) were in the HD group.

### 3.1. Patient Characteristics

The median GA was identical in both groups at 28 weeks (IQR 27–30 and 26–30 in LD and HD groups, respectively). Mean birthweight was 1141 g (SD 380) and 1114 g (SD 352) in the LD and HD groups, respectively. There were no significant differences in some common perinatal factors such as antenatal steroid use, chorioamnionitis, mode of delivery, or one and five min Apgar scores between groups (Table 1). Similarly, when we evaluated some common neonatal/postnatal risk factors, such as gender, post-natal steroids, PDA, and PDA surgical ligations, we saw no significant differences between the groups. However, the HD group did have a significantly increased prevalence of culture positive sepsis (LD = 0%, HD = 5.6%, *p* < 0.01).

### 3.2. Respiratory Outcomes

The mean total duration of mechanical ventilation up to 36 weeks PMA or discharge was 205.5 (SD = 400.4) hours for the entire cohort, and 236.1 (SD = 436.4) and 143.4 (SD = 307.9) hours for the LD and HD groups, respectively (*p* = 0.044). The adjusted linear regression model showed that the HD group had 91.4 fewer hours of mechanical ventilation than the low-dose group (*p* = 0.018) (Table 2).

The majority of patients were mechanically ventilated at some point within the first 28 DOL; 46.5% (*n* = 127) of all cohort infants; 40% (*n* = 73) and 60% (*n* = 54) of infants in the LD and HD groups, respectively (*p* = 0.175). Indications for mechanical ventilation were not tracked and were left up to the clinical team. This unadjusted estimate suggested increased need for mechanical ventilation in the HD group; however, adjusted logistic regression modeling revealed a lack of evidence to conclude that the need for mechanical ventilation in the first 28 DOL differed by caffeine groups (*p* = 0.161) (Table 3). Similarly, adjusted linear regression modeling for duration of non-invasive support failed to show a difference between the groups (*p* = 0.990) (Table 2).

Overall, in the entire cohort, data for BPD analysis was available in 233 patients, of which 46.8% (*n* = 109), 31.8% (*n* = 74), and 21.5% (*n* = 50) had none, mild, or moderate/severe BPD, respectively. When looking at the overall incidence of BPD, including all severity subtypes, it was similar for both groups; 54% (*n* = 87) and 50% (*n* = 37) of infants in the LD and HD groups, respectively (*p* = 0.501). Adjusted logistic regression modeling did not show statistical evidence to conclude that BPD differed by caffeine groups (*p*-value = 0.147) (Table 3). When we dichotomized BPD into none/mild and moderate/severe groups, unadjusted measures showed significantly less moderate/severe BPD in the HD group; 25% (*n* = 41) in the LD group and 12.2% (*n* = 9) in the HD group (*p* = 0.018). The adjusted logistic regression model showed a statistically significant decrease as well (OR 0.37 (95% CI: 0.14, 0.97; *p* = 0.042)) (Table 3).

### 3.3. Additional Neonatal Outcomes and Comorbidities

Significant apneic episode frequency until 36 weeks PMA did not differ between the groups (*p* = 0.518, adjusted linear regression model) (Table 2). The mean length of stay was 76.4 (SD 45.0) days for the entire cohort, and 78.6 (SD 46.4) and 72.0 (SD 42.0) days for the low and high-dose groups, respectively (*p* = 0.254, independent samples *t*-test). Major comorbidities measured included ROP, severe ROP, NEC, and death. Mortality as well as measured comorbidities remained unaffected (Table 4).

## 4. Discussion

In this retrospective single center study, caffeine citrate started early (within 24 h of life) with a loading dose of 20 mg/kg, and a maintenance dose of 10 mg/kg/day was associated with a statistically significant reduction in the total duration of mechanical ventilation up to 36 wk PMA or discharge compared to a maintenance dose of 6 mg/kg/day, as well as a decrease in moderate/severe BPD.

Although several centers are beginning to adopt high-dose, 10 mg/kg caffeine citrate (5 mg/kg caffeine base) maintenance dosing as standard of care, there are still many NICUs globally that adhere to a lower dose of caffeine on a daily basis. Investigators have previously reported an association between initiation of caffeine early versus later in life and a shorter duration of mechanical ventilation [12,13]. Dobson et al. [13] also showed that early caffeine therapy led to shorter duration of mechanical ventilation (mean difference of six days; *p* < 0.001). There is a gap in the literature, however, for the combination of early and high-dose caffeine which our study addresses. The reduction in duration of mechanical ventilation and moderate/severe BPD seen in our study may be due to the combination of effects of use of higher dosing as well as earlier initiation. It is possible that the exact time of administration of caffeine, or the dose alone, may have an effect; however, due to the variability of practice in caffeine dose and timing of administration prior to initiating the CPG, we could not adequately or statistically control for this in our analysis. The focus for the CPG was the change to a higher dose of caffeine citrate but emphasized giving the dose early (within 24 h). This is the reason why we combined the outcomes of HD and early administration of caffeine.

In our study, infants in the early HD group had 91.4 (95% CI: −166.6, −16.1; adjusted linear regression) less hours of mechanical ventilation up to 36 weeks PMA or discharge as compared with the LD group. We postulate that this reduction in duration of mechanical ventilation may be due to the role of higher doses of caffeine in the peri-extubation phase [15]. In our cohort, there was no difference in the need for mechanical ventilation in the first 28 days of life, and this mostly accounts for the many variables associated with the care of preterm infants early in life and the likely accumulative benefit on long-term use of caffeine. There was no difference between groups in the last pH and pCO2 prior to extubation (Table 1), suggesting that the difference in duration of mechanical ventilation was not due to allowing for more permissive hypercapnia or minimization of ventilatory settings while intubated. The lack of difference between caffeine groups in the duration of non-invasive mechanical ventilation may be due, in part, to our institutional guidelines which recommend continuing CPAP to 32 wk PMA for optimal growth; this may also be the reason there was no difference in overall BPD incidence i.e., any BPD vs. no BPD. However, when analyzing the differences in the combined outcome of moderate and severe BPD, we did see a statistically significantly reduction in the prophylactic high-dose group. Literature has shown that the more severe forms of BPD prognosticate worse pulmonary function in childhood [24] and worse long-term neurological outcomes [25,26,27]. Hence, we hypothesize that high-dose prophylactic caffeine may potentially lead to an improvement in these long-term pulmonary and neurological outcomes as well.

We studied the comorbidities of ROP, NEC, and death, in which we did not see any significant difference. We did, however, note a difference in the culture positive sepsis, with the HD group having a higher rate, which was unexpected. There were no changes in antimicrobial usage guidelines during the two epochs. When evaluating our rates of infection prior to our study period, and comparing them to the HD rates, they were both found to be similar. Therefore, we attribute this finding to an unusually low rate of sepsis during our study period where most infants in the LD were included. A common concern regarding caffeine therapy is tachycardia, the overall incidence based on one post authorization study is low at 2.3% [28]. While our database did not allow us to accurately capture data on the period of tachycardia, we did look at infants who had premature discontinuation of caffeine therapy or a decrease in dosing (*n* = 5), and none of them were attributed to tachycardia. There has also been concern regarding an increase in IVH with significantly higher dosing of caffeine use than studied here. One study evaluating the prophylactic use of high-dose caffeine (total dose of 80 mg/kg over 36 h), compared to standard dose, showed that patients in the high-dose group had a higher incidence of cerebellar hemorrhage (36% vs. 10%) along with increased tone and abnormal movements. Our study was not powered to analyze outcomes of IVH, as not all infants received imaging for IVH. However, in other clinical trials within the ranges of caffeine dose in our study, there were no noted differences in seizures or brain effects [10].

Limitations of this study include the retrospective and observational design which prevents us from concluding whether there were definitive causal effects of prophylactic high-dose caffeine on the respiratory outcomes. Additionally, data for BPD outcome analysis was not present in our entire study population and we were not able to adequately capture data on tachycardia and IVH. Our study has several strengths; the sample size was large enough to provide estimates of association for our primary outcome. We looked at certain factors which allowed us to account for changes in practices such as permissive hypercapnia. Additionally, we adjusted for multiple potential confounders which were based on literature-based associations with the outcomes of interest.

## 5. Conclusions

This study adds evidence indicating that the initiation of early caffeine citrate at maintenance doses of 10 mg/kg/day may reduce the duration of mechanical ventilation and decrease the incidence of moderate and severe BPD. Considering the limited therapies available for reducing BPD and its severity, optimizing the timing and dose of caffeine therapy may be a very feasible and safe approach to decreasing the burden of neonatal and childhood respiratory morbidity. However, the data on the benefit of both early and high maintenance dose caffeine usage are retrospective. We believe a randomized controlled trial of high-dose caffeine prophylaxis to prevent neonatal morbidities, such as BPD, is required to conclusively support the routine use of high-dose caffeine as a preventative therapy and to ensure its safety in preterm infants.

## Figures and Tables

**Table 1:** Baseline characteristics of study groups.

Baseline Characteristic	Low-Dose (*n* = 183)	Early High-Dose (*n* = 90)	*p*-Value
Gender, *n* (%)			0.467
Male	96 (52.5)	43 (47.8)	
Female	87 (47.5)	47 (52.2)	
Gestational age (weeks), Median (IQR)	28.0 (26.0–30.0)	28.0 (27.0–30.0)	0.922
Birth weight (grams), Mean (SD)	1141.3 (380.9)	1114.4 (352.3)	0.575
Maternal age (years), Mean (SD)	29.1 (6.7)	27.7 (6.5)	0.118
Gravida, median (IQR)	2 (1–4)	2 (1–4)	0.266
Para, median (IQR)	1 (0–2)	1 (0–2)	0.210
Singleton, *n* (%)	141 (77.1)	71 (78.9)	0.732
Apgar score, *n* (%)			
1 min			0.412
0–3	74 (40.4)	34 (37.8)	
4–6	56 (30.6)	23 (25.6)	
7–10	53 (29.0)	33 (36.7)	
5 min			0.387
0–3	19 (10.4)	13 (14.4)	
4–6	55 (30.1)	21 (23.3)	
7–10	109 (59.6)	56 (62.2)	
Mode of delivery, *n* (%)			0.201
Vaginal	70 (38.3)	27 (30.3)	
Cesarean	113 (61.8)	62 (69.7)	
Maternal chorioamnionitis, *n* (%)	21 (12.3)	5 (5.7)	0.094
Antenatal steroids, *n* (%)	135 (83.3)	78 (89.7)	0.176
Post–natal steroids, *n* (%) †	7 (3.8)	3 (3.3)	1.000
PDA, *n* (%)	82 (55.0)	29 (46.0)	0.230
PDA Surgical ligation, *n* (%) †	23 (28.1)	4 (13.8)	0.140
Culture positive sepsis, *n* (%) †	0 (0.0)	5 (5.6)	0.004
Last pH prior to extubation, Median (IQR)	7.34 (7.31–7.39)	7.34 (7.31–7.39)	0.888
Last pCO2 prior to extubation, Mean (SD)	42.2 (8.2)	40.4 (6.6)	0.205

IQR = interquartile range; SD = standard deviation; † used Fisher’s exact.

**Table 2:** Linear Regression models for outcomes of interest (prophylactic high-dose vs. low-dose).

Outcomes of Interest	Estimate	95% CI	*p*-Value
Duration of mechanical ventilation up to 36 wk PMA or discharge ^1^	−91.4	−166.6, −16.1	0.018
Duration on non-invasive respiratory support up to 36 wk PMA or discharge ^2^	0.18	−88.8, 89.1	0.997
Significant apneas up to 36 wk PMA or discharge ^2^	−4.93	−19.95, 10.08	0.518

^1^ adjusted for GA, gender, postnatal steroids and PDA. ^2^ adjusted for GA.

**Table 3:** Logistic Regression models for outcomes of interest (prophylactic high-dose vs. low-dose).

Outcomes of Interest	OR	95% CI	*p*-Value
Need for mechanical ventilation in the first 28 days of life ^1^	0.55	0.24, 1.27	0.161
Any BPD ^2^	0.53	0.22, 1.25	0.147
Mod/Severe BPD ^2^	0.37	0.14, 0.97	0.042

OR = Odds Ratio; CI = Confidence interval; ^1^ adjusted for GA, mode of delivery, gender, and PDA. ^2^ adjusted for GA, gender, chorioamnionitis.

**Table 4:** Major comorbidities by caffeine group.

Major Comorbidity	Low-Dose (*n* = 183)	Early High-Dose (*n* = 90)	*p*-Value
ROP, *n* (%)	36 (20.9)	10 (11.8)	0.071
NEC, *n* (%) †	11 (6.0)	6 (6.7)	0.796
Death, *n* (%) †	12 (6.7)	5 (5.6)	0.797

† used Fisher’s exact.

## Data Availability

The data presented in this study are available on request from the corresponding author.
